# *Gentiana sino-ornata* Balf. f. ethyl acetate extract alleviates high-fat diet- and tetracycline-induced liver injury in mice: insights from serum pharmacochemistry, network pharmacology, and experimental validation

**DOI:** 10.3389/fphar.2026.1923448

**Published:** 2026-09-08

**Authors:** Mi Zhang, Zhaobin Xia, Qingyun Gu, Lincheng Ye, Chaoxi Chen

**Affiliations:** 1 College of Animal and Veterinary Sciences, Southwest Minzu University, Chengdu, China; 2 College of Ecology and Agriculture, Sichuan Minzu College, Kangding, China; 3 Key Laboratory of Ecological Protection and Characteristic Industry Cultivation in Hengduan Mountain Area at Sichuan Minzu College, Kangding, China; 4 College of Animal Science and Technology, Xizang Vocational Technical College, Lhasa, China

**Keywords:** DILI, *Gentiana sino-ornata* Balf. f., gut microbiota, NAFLD, network pharmacology, PI3K/Akt/FoxO1 signaling pathway

## Abstract

**Background:**

Nonalcoholic fatty liver disease (NAFLD) is one of the most prevalent chronic liver diseases worldwide, and the associated metabolic dysfunction may increase susceptibility to drug-induced liver injury (DILI). This study aimed to evaluate the hepatoprotective effects of the *Gentiana sino-ornata* Balf. f. ethyl acetate extract (GSEA) in mice exposed to a high-fat diet (HFD) and tetracycline.

**Methods:**

The antioxidant and anti-inflammatory activities of GSEA were evaluated *in vitro* and *in vivo*. Subsequently, the blood-absorbed ingredients of GSEA were characterized using UHPLC-HRMS, followed by network pharmacology analysis and molecular docking to elucidate the potential mechanisms underlying its hepatoprotective effects. Finally, the predicted mechanisms were experimentally validated in a mouse model of HFD- and tetracycline-induced liver injury.

**Results:**

GSEA exhibited significant antioxidant and anti-inflammatory activities. Twenty-five blood-absorbed ingredients were tentatively identified as potentially bioactive, and the PI3K/Akt/FoxO1 signaling pathway was predicted as a pathway of GSEA against liver injury. Molecular docking further demonstrated favorable binding interactions between AKT1 and several major constituents, including sweroside, loganic acid, swertiamarin, and gentiopicroside. *In vivo* experiments showed that GSEA markedly alleviated HFD- and tetracycline-induced liver injury by suppressing the expression of inflammation- and glucolipid-metabolism-related genes, attenuating hepatic inflammation, modulating the gut microbiota, and partially regulating the PI3K/Akt/FoxO1 signaling pathway.

**Conclusion:**

Collectively, these findings provide mechanistic evidence supporting the potential application of GSEA as a natural therapeutic agent for the prevention or treatment of liver injury associated with metabolic dysfunction and tetracycline exposure.

## Graphical Abstract

**Figure:**
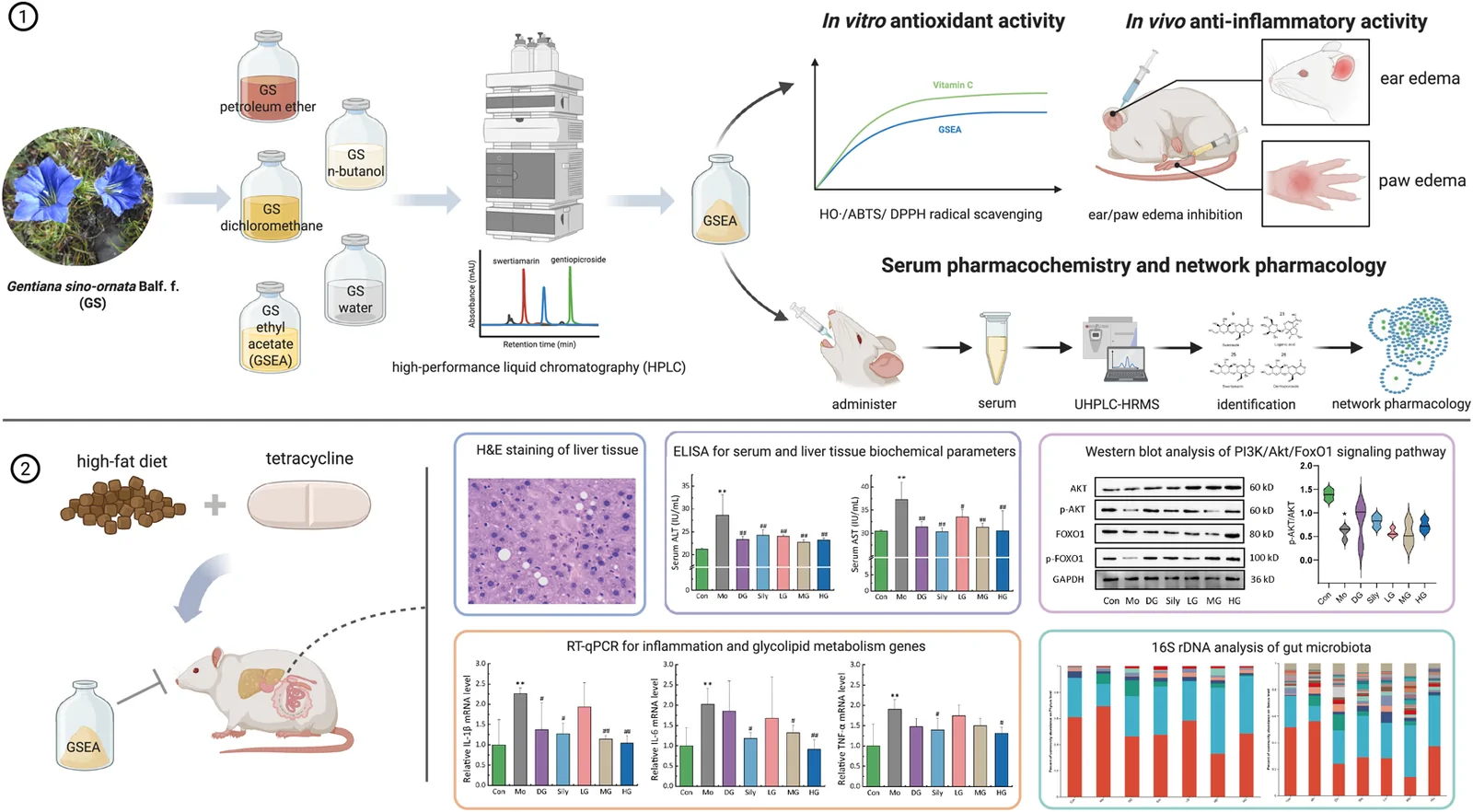
BioRender. Xia, Z. (2026) https://BioRender.com/a7q66hi.

## 1 Introduction

As a central organ in metabolic regulation, the liver performs essential functions in xenobiotic detoxification, energy homeostasis, and immune regulation. Nevertheless, liver diseases account for more than two million deaths worldwide each year, corresponding to approximately 4% of global mortality, or one in every 25 deaths (Devarbhavi et al., 2023). Nonalcoholic fatty liver disease (NAFLD), now referred to as metabolic dysfunction-associated steatotic liver disease (MASLD), is one of the most prevalent chronic liver disorders worldwide. NAFLD has historically been estimated to affect approximately 25% of the global adult population and has become an increasingly important cause of end-stage liver disease and liver transplantation in Europe and North America (Devarbhavi et al., 2023). Long-term consumption of a high-fat diet (HFD) is a major risk factor for its development and progression, as it promotes excessive hepatic lipid accumulation, oxidative stress, and inflammatory responses, ultimately leading to hepatocellular injury (Larter et al., 2012; Wu et al., 2022; Lv et al., 2024). Drug-induced liver injury (DILI) is another important cause of liver disease. In China, its annual incidence has been estimated at 23.8 cases per 100,000 individuals (Shen et al., 2019). Accumulating evidence suggests that pre-existing NAFLD may alter susceptibility to DILI, although the extent of this effect varies depending on the causative drug and the severity of the underlying metabolic disorder (Bessone, 2018; Lammert et al., 2019). Some drugs that exhibit limited hepatotoxicity under metabolically healthy conditions may aggravate hepatic steatosis and injury in obese or steatotic animal models (Bessone, 2018; Sun et al., 2018; Li et al., 2025; Walsh et al., 2025). Antibiotics and antituberculosis agents are among the drug classes frequently implicated in DILI (Gu et al., 2023). Tetracyclines have been widely used in food-producing animals because of their broad-spectrum antibacterial activity and relatively low cost (Xu et al., 2020; Hunge et al., 2022). Widely used across many countries and regions, tetracyclines rank as the most frequently used, comprising 22% of total antibiotic consumption in Germany, 20% in the European Union and Switzerland, and 18% in China (Xu et al., 2020). High-dose or prolonged tetracycline exposure has been associated with mitochondrial dysfunction, oxidative stress, cytoplasmic vacuolization, sinusoidal dilation, and hepatocellular necrosis (Shabana et al., 2012; López-Pascual et al., 2024; Yao et al., 2024). HFD-induced metabolic dysfunction may modify, and under certain conditions increase, susceptibility to xenobiotic-induced liver injury (Li et al., 2026).

Traditional herbal medicines act as an important source of potential therapeutic agents for liver diseases because of their diverse phytochemical constituents and capacity to act on multiple biological targets. *Silybum marianum* L. has a long history of use in the management of hepatic and biliary disorders (Stolf et al., 2017). Silymarin, its principal bioactive complex, has been reported to alleviate NAFLD and steatohepatitis by attenuating oxidative stress and hepatic lipid accumulation while preserving mitochondrial function (Federico et al., 2017). Likewise, *Glycyrrhiza* species are widely used in traditional medicine to treat liver disorders. Diammonium glycyrrhizinate (DG), a glycyrrhizin derivative, is used clinically as a hepatoprotective agent and has shown beneficial effects against nonalcoholic steatohepatitis and DILI, largely through its antioxidant and anti-inflammatory activities (Jin et al., 2014; Gong et al., 2022).

Species of the genus *Gentiana* are widely distributed and have long been used in traditional Chinese and ethnomedicine. Their reported pharmacological activities include anti-inflammatory, antioxidant, and metabolic regulatory effects, which may contribute to their hepatoprotective potential (Jiang et al., 2021). Gentiopicroside and swertiamarin are the representative bioactive secoiridoid glycosides widely found in *Gentiana* species (Valenta Šobot et al., 2024; Yu et al., 2025). Gentiopicroside exhibits a broad range of pharmacological activities, including the suppression of inflammation and oxidative stress, regulation of lipid metabolism, and amelioration of NAFLD-related pathological changes (Wu et al., 2017; Li et al., 2018; Xiao et al., 2022). Similarly, swertiamarin has been reported to attenuate fructose-induced NAFLD and HFD-induced obesity by reducing hepatic oxidative stress and inflammation and limiting lipid accumulation (Yang et al., 2019; Xu et al., 2021). Nevertheless, although *Gentiana sino-ornata* Balf. f. (GS) is used in ethnomedicine, its chemical basis and hepatoprotective properties remain insufficiently characterized. Previous studies have predominantly focused on the pharmacological effects of individual constituents. In the present study, major constituents characteristic of *Gentiana* species was selected as quantitative chemical markers to guide the selection of an ethyl acetate fraction enriched in these compounds. Serum pharmacochemistry and network pharmacology were subsequently integrated with *in vivo* experiments using a combined HFD- and tetracycline-induced liver injury model of metabolic dysfunction and drug-exacerbated liver injury. This model was designed to mimic, to some extent, the clinical context in which drug exposure aggravates hepatic injury in individuals with pre-existing metabolic dysfunction. Using this integrated approach, we investigated the hepatoprotective effects of the multicomponent ethyl acetate fraction of *Gentiana* sino-ornata and its potential mechanisms involving multiple targets and signaling pathways, thereby providing experimental evidence for its further pharmacological development and application.

## 2 Materials and methods

### 2.1 Plant material, chemical agents, and biological materials

Whole plants of GS were collected from Danqing Yakwang Shenshan, Seda County, Garze Tibetan Autonomous Prefecture, at an altitude of 4,010 m (latitude: 30°12′46″N, longitude: 101°40′30″E) in November 2021. A voucher specimen was deposited in the Laboratory of Veterinary Pharmacology and Toxicology at the College of Animal and Veterinary Sciences. Gentiopicroside (purity ≥98%) and swertiamarin (purity ≥98%) were purchased from DeSiTe Biological Technology Co., Ltd (Chengdu, China). Commercial assay kits for triglyceride, total cholesterol, and malondialdehyde were obtained from Nanjing Jiancheng Bioengineering Institute (Nanjing, China). HiScript Q RT SuperMix for qPCR and Taq Pro Universal SYBR qPCR Master Mix test kits were purchased from Vazyme Biotech Co., Ltd. (Nanjing, China). Antibodies against protein kinase B (Akt), phosphorylated-Akt (p-Akt), forkhead box O1 (FoxO1), phosphorylated-FoxO1 (p-FoxO1, Ser256), and glyceraldehyde-3-phosphate dehydrogenase (GAPDH), and the goat anti-rabbit IgG (H + L) secondary antibody were purchased from Beyotime Biotechnology Co., Ltd (Shanghai, China).

### 2.2 Animals

Six-week-old SPF-grade male Kunming mice were obtained from Chengdu Dossy Experimental Animal Co., Ltd. [SCXK (Sichuan) 2020-030]. All mice were housed in a temperature- and humidity-controlled animal facility with *ad libitum* access to food and water and acclimatized for a week before the experiment. All animal procedures were approved by the Animal Ethics Committee of Southwest Minzu University and performed in accordance with the regulations and guidelines of the Institutional Animal Care and Use Committee.

### 2.3 Preparation of different GS fractions and quantitative analysis of the main constituents

Two kilograms of naturally dried and powdered GS was extracted three times with 10 L of 55% (v/v) ethanol at a solid-to-liquid ratio of 1:5 (w/v). Each extraction was performed by ultrasound-assisted extraction for 45 min. The extracts were pooled and concentrated under reduced pressure, then suspended in deionized water and successively partitioned with petroleum ether, dichloromethane, ethyl acetate, and n-butanol. Each fraction was concentrated to remove the organic solvent and then dried to obtain the corresponding GS fraction. For quantitative analysis, 10 mg of the partitioned GS extracts was dissolved in 10 mL of deionized water, passed through a 0.22 μm filter, and analyzed by high-performance liquid chromatography (HPLC). Gentiopicroside and swertiamarin were quantified using external standard calibration curves. HPLC analysis was performed on a PerkinElmer Altus10 system equipped with a Thermo Scientific Acclaim 120 C_18_ reverse-phase column (4.6 × 250 mm, 5 μm). The setup involved a 10 μL injection volume, a column temperature of 25 °C, and a detection wavelength of 254 nm. The mobile phase consisted of methanol (A) and 0.2% phosphoric acid in water (B). The gradient elution program was as follows: 0–5 min, 30% A; 5.01–10 min, 30%–35% A; 10.01–15 min, 35% A; 15.01–20 min, 35%–50% A; 20.01–25 min, 50% A; 25.01–30 min, 50%–30% A.

### 2.4 *In vitro* antioxidant activity of GS ethyl acetate extract (GSEA)

The radical-scavenging activities were evaluated using ABTS/DPPH/·OH assays *in vitro*, following the reported methods with several modifications (Wu et al., 2015; Shang et al., 2018; Xia et al., 2025). GSEA solutions at different concentrations were tested, with vitamin C (VC) used as the positive control. The free radical scavenging ability was calculated by the formula: hydroxyl radical scavenging activity (%) = [1 *−* (A_1_ *−* A_2_)/A_0_] × 100%, where A_0_, A_1_ and A_2_ were the absorbance of the control group (distilled water or absolute alcohol instead of the sample), the absorbance of the test sample mixed with the reaction solution and the absorbance of the sample, respectively.

For the ABTS radical scavenging assay, the ABTS radical (ABTS^+^) was produced by mixing 800 µL of ABTS solution (7 mM) with 200 µL of potassium persulfate aqueous solution (15 mM). The mixture was reacted with 200 µL of gradient GSEA solutions and VC. After incubation at 20 °C for 6 min, the absorbance was measured at 734 nm using distilled water as the reference blank. For the DPPH radical scavenging assay, 100 μL of 0.1 mM DPPH solution in ethanol was prepared and mixed with 100 μL of gradient GSEA solutions and VC. The mixture was placed at room temperature. After incubation for 30 min, the mixture was determined at 517 nm against ethanol. For hydroxyl radical scavenging ability, 200 µL of 9 mM FeSO_4_ aqueous solution, 200 µL of 9 mM salicylic acid-ethanol solution, and 200 µL of 3.8 mM H_2_O_2_ were added to a 5 mL glass tube containing 200 µL of gradient GSEA solutions and VC. The mixture was incubated at 37 °C for 30 min, and the absorbance at 510 nm was recorded with a spectrophotometer.

### 2.5 *In vivo* anti-inflammatory activity of GSEA

A total of 35 male Kunming mice, weighing 18–22 g, were randomly assigned to five groups (n = 7): a model group (Mo), which received distilled water; low-, medium-, high-dose of GSEA groups (LG, 380 mg·kg^−1^·bw^−1^; MG, 750 mg·kg^−1^·bw^−1^; HG, 1,500 mg·kg^−1^·bw^−1^, respectively); and an aspirin positive-control group (ASP, 600 mg·kg^−1^·bw^−1^). Each treatment was administered orally *via* a gastric tube once daily for seven consecutive days, and the inflammatory model was established after the final administration. For the xylene-induced ear edema model, 30 μL of xylene was applied to the right ear 1 h after the final administration; an equal volume of normal saline was applied to the left ear as the contralateral control. At the end of the challenge period, both ears were excised, and circular sections 9.0 mm in diameter were punched from corresponding sites and weighed. For the ovalbumin-induced paw edema model, mice received a subcutaneous injection of 100 μL of 10% ovalbumin into their right hind paw, and the left paw was injected with an equal volume of normal saline as a control. One hour after ovalbumin injection, the mice were euthanized by cervical dislocation, and both hind paws were excised and weighed. The edema inhibition rate was calculated using the equation: Ear/paw edema inhibition rate (%) = [(A_0_ − A_1_)/A_0_] × 100%. A_0_ represents the average tissue weight (ear or paw) in the Mo group, whereas A_1_ denotes the average tissue weight in the treatment group.

### 2.6 Chemical constituents and blood-absorbed ingredients of GSEA

Blank serum samples (Serum-Blank) were collected on day 0 before GSEA administration. The mice were then orally administered GSEA at 938 mg kg^−1^·bw^−1^ once daily for a week. Serum was collected 30 min after the final dose. UHPLC-HRMS (Ultra-high-performance liquid chromatography coupled with high-resolution mass spectrometry) was used to characterize the chemical constituents of GSEA and to compare the profiles of Serum-Blank and Serum-GSEA. Compound structural identification was achieved by integrating high-resolution mass spectral information with the compounds registered in established compound databases. Using an integrative approach combining chemical analysis and quality control, a compound was classified as a candidate bioactive and absorbed into the blood following oral administration if it appeared in the GSEA and its peak intensity in the Serum-GSEA was at least threefold higher than that in the Serum-Blank. The blood-absorbed ingredients of GSEA were determined by comparative analysis and confirmed using consensus-based selection criteria. Chromatographic separation was performed using an ACQUITY UPLC system equipped with an ACQUITY UPLC HSS T3 column (2.1 × 100 mm, 1.8 μm, Waters) maintained at 35 °C. The mobile phase consisted of (A) 0.1% formic acid in water and (B) 0.1% formic acid in acetonitrile. The flow rate was 0.3 mL min^−1^, and the injection volume was 4 μL. Mass spectrometry was performed in both positive- and negative-ion modes using full MS-ddMS^2^ detection; the crucial parameters were as follows: a spray voltage of 3.8 kV for positive-ion mode and −3.0 kV for negative-ion mode, and sheath and auxiliary gas flow rates set at 45 and 20 Arb, respectively, a capillary temperature of 320 °C, and a probe heater temperature of 370 °C.

### 2.7 Network pharmacology

A network pharmacology analysis was constructed for the screened blood-absorbed ingredients. In brief, SwissTargetPrediction (http://www.swisstargetprediction.ch/) was employed to predict potential targets for the identified blood-absorbed ingredients, and a “component-target” network was created using Cytoscape 3.9.1. NAFLD-related disease targets were retrieved from the databases GeneCards (https://www.genecards.org/), OMIM (https://www.omim.org/), DisGeNET (https://www.disgenet.org/), and TTD (http://www.db.idrblab.net/ttd/). Potential therapeutic targets for GSEA-based treatment of NAFLD were identified by intersecting disease-related targets with the predicted targets of blood-absorbed ingredients. The common targets were subsequently analyzed as a protein-protein interaction (PPI) network with STRING 12.0 (https://string-db.org/) and then imported into Metascape (https://metascape.org/) for Gene Ontology (GO) and Kyoto Encyclopedia of Genes and Genomes (KEGG) pathway enrichment analysis. A visualization of the top 20 pathways with enriched targets was created, and a “component-target-pathway” network was established to delve deeper into the key targets. Molecular docking, performed with AutoDock Vina, evaluated the binding affinity between the targets and the key blood-absorbed ingredients, and the results were visualized using PyMOL 2.4.0.

### 2.8 Establishment of the liver injury model and experimental treatment

Fifty-six Kunming mice were randomly assigned into seven groups (n = 8): a control (Con), model (Mo), two positive-control groups treated with Silymarin (Sily, 100 mg kg^−1^·bw^−1^) and diammonium glycyrrhizinate (DG, 100 mg kg^−1^·bw^−1^), and three GSEA-treated groups receiving low, medium, or high doses of GSEA (LG, 469 mg kg^−1^·bw^−1^; MG, 938 mg kg^−1^·bw^−1^; HG, 1875 mg kg^−1^·bw^−1^). Beginning on day 1, mice in the Con group received normal saline intraperitoneally, whereas mice in all other groups received tetracycline (100 mg kg^−1^·bw^−1^) at 5-day intervals during the first 30 days. From day 31 through day 44, mice in the treatment groups were administered their respective drugs orally, while the Con and Mo groups received sterile distilled water. During the period of the experiment, mice in the Con group were fed a standard chow diet, whereas those in all other groups were fed a high-fat diet (HFD) (66.5% standard chow, 10% lard, 1% choline bitartrate, 2.5% cholesterol, and 20% sucrose). On day 44, blood was collected from the retro-orbital venous plexus for serum separation. The mice were then euthanized by cervical dislocation, and fecal samples and liver tissues were collected for subsequent analyses.

### 2.9 Histological assessment

Liver samples were rinsed using pre-cooled PBS and fixed overnight in 4% paraformaldehyde. The preserved liver tissues were rinsed with deionized water, dehydrated in a graded series of ethanol (30%, 50%, 70%, 80%, 95%, and 100%), treated with xylene, and subsequently immersed in paraffin at 60 °C. Following fixation, the tissues needed to ensure the soft tissue was sufficiently supported for cutting into thin sections at the desired thickness, usually 4–5 µm. The sections were then deparaffinized, rehydrated, and stained with hematoxylin and eosin (H&E) before being analyzed using an Olympus CH2 microscope (Olympus, Japan).

### 2.10 Serum and liver tissue biomarkers

A glucose meter was used to measure blood glucose levels, while serum alanine aminotransferase (ALT) and aspartate aminotransferase (AST) were determined using a microplate-based colorimetric assay. According to manufacturers’ instructions, serum and hepatic concentrations of total cholesterol (TC), triglycerides (TG), and serum malondialdehyde (MDA) were quantified using commercial kits from the Nanjing Jiancheng Bioengineering Institute.

### 2.11 Quantitative real-time polymerase chain reaction (qRT-PCR) assay

Liver tissue RNA was extracted with TRIzol reagent following the manufacturer’s instructions. Total RNA was then converted to cDNA using the HiScript II Q RT SuperMix for qPCR (+ gDNA wiper) Kit from Vazyme Biotech Co., Ltd, China. For qRT-PCR amplification, cDNA template, primers (listed in Supplementary Table S1), 2 × Taq Pro Universal SYBR qPCR Master Mix provided by Vazyme Biotech Co., Ltd, China, and RNase-free ddH_2_O were employed. The qRT-PCR started with an initial denaturation at 95 °C for 60 s, followed by 40 cycles of amplification at 95 °C for 10 s and 60 °C for 30 s, an extension phase at 60 °C for 60 s, and finished with a final denaturation at 95 °C for 15 s. The 2^−ΔΔCt^ method was employed to calculate fold changes in expression, with glyceraldehyde-3-phosphate dehydrogenase (GAPDH) as the endogenous control.

### 2.12 Western blotting

Using ice-cold RIPA buffer with 1 mM protease and phosphatase inhibitors, total protein was extracted from 50 mg frozen liver tissue by homogenization, and the protein concentration was measured using a commercial BCA kit. Each sample, containing 30 μg protein, was denatured by boiling in loading buffer for 5 min before being loaded onto a 10% SDS-polyacrylamide gel. Electrophoresis was conducted at 80 V for the stacking gel and 180 V for the running gel using SDS running buffer. Proteins were subsequently transferred to a PVDF membrane with a pore size of 0.22 μm. Initially, the membranes underwent a 2-h incubation with a 5% non-fat dry milk solution to block nonspecific binding, followed by an overnight incubation at 4 °C with primary antibodies (diluted to 1:1,000). Following this, species-specific secondary antibodies (diluted to 1:1,000) were applied for 1 h at room temperature. Protein quantification was achieved using a chemiluminescent imaging system and ECL reagents (MiniChemi, China). Densitometric analysis with ImageJ (v1.5, National Institutes of Health, Bethesda, MD, United States) was used to quantify band intensities, and GAPDH, a housekeeping gene, served as an internal control to normalize the target gene expression.

### 2.13 Microbial diversity and composition

Bacterial DNA from fecal samples was extracted, and its purity and concentration were assessed by measuring absorbance at 260 nm with a NanoDrop Spectrophotometer (Waltham, MA, United States). The universal primers 338F (5′-ACT​CCT​ACG​GGA​GGC​AGC​AG-3′) and 806R (5′-GGACTACHVGGGTWTCTAAT-3′) were used to PCR-amplify the V3-V4 hypervariable region of the 16S ribosomal DNA gene. Amplicons were extracted, purified, and quantified using agarose gel electrophoresis. The qualified amplicons were pooled into equimolar fractions for paired-end sequencing on an Illumina HiSeq2500 system (Illumina, San Diego, CA, USA). Once assembled and filtered, the clean sequences were categorized into operational taxonomic units (OTUs) at a 97% nucleotide threshold. The final annotation results had confidence intervals ranging from 0.7 to 1, and the bioinformatics analysis was conducted using the obtained sequencing data.

### 2.14 Statistical analysis

The data are presented as mean ± SEM and analyzed using SPSS 25. One-way ANOVA and Duncan’s multiple comparison test were used to assess group differences. Data visualization was performed in Origin 2021, with a significance threshold of *P* < 0.05.

## 3 Results

### 3.1 Antioxidant and anti-inflammatory activities of GSEA

**FIGURE 1:**
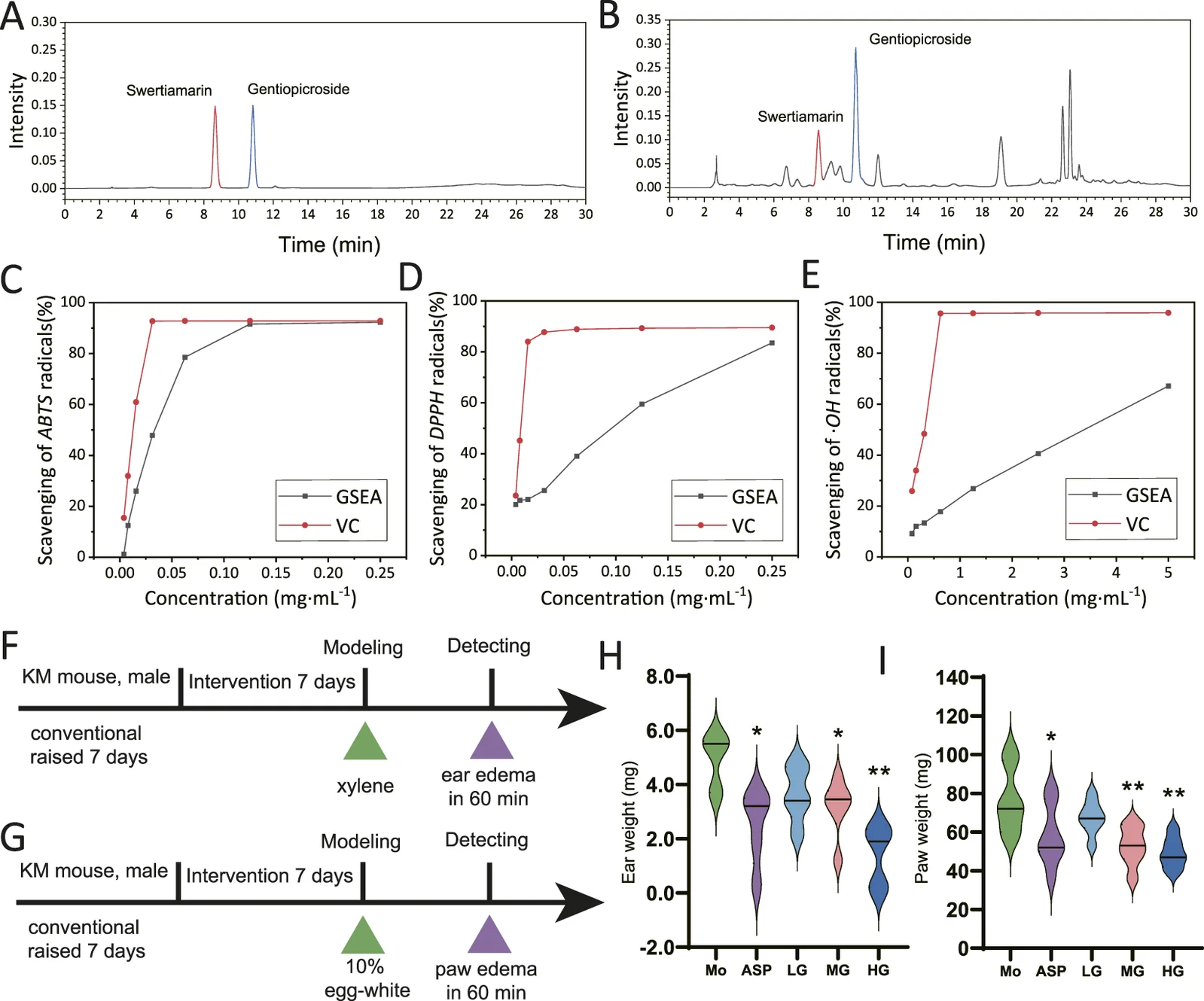
Antioxidant and anti-inflammatory characteristics of GSEA. **(A,B)** HPLC chromatograms of gentiopicroside and swertiamarin reference standards, together with a chromatogram for GSEA. **(C–E)** Antioxidant potential was evaluated using ABTS, DPPH, and hydroxyl radical scavenging assays. **(F–I)** Schematic diagram and quantitative analysis of ear and paw swelling. Data are presented as mean ± SEM (n = 5). ^*^ *P* < 0.05, ^**^ *P* < 0.01 vs. Mo group.

Species of the genus *Gentiana* are rich in terpenoids, particularly secoiridoid glycosides, and have been traditionally used to treat a range of conditions, including rheumatoid arthritis and hepatic cirrhosis. Its constituents, such as gentiopicroside and swertiamarin, are known for their prominent anti-inflammatory, antioxidant, and hepatoprotective properties. To enrich these constituents, GS extracts were prepared and partitioned using different organic solvents. The concentrations of gentiopicroside and swertiamarin were subsequently quantified using HPLC. The ethyl acetate fraction contained the highest levels of swertiamarin and gentiopicroside, with concentrations of 19.70 mg g^−1^ and 49.73 mg g^−1^, respectively. Figures 1A,B present the HPLC chromatograms of the reference standards and sample extracts, and Supplementary Text S1 confirms methodological validation, showing that the established HPLC method has excellent specificity, precision, stability, and reproducibility.

According to the antioxidant capacity assessments, GSEA demonstrated potent ABTS free radical scavenging activity, similar to that of VC at high concentrations. At a concentration of 250 μg/mL, GSEA had an ABTS radical-scavenging rate of 92.30%, while VC achieved 92.85% (Figure 1C). Similarly, GSEA showed a DPPH radical-scavenging capacity comparable to VC, achieving scavenging rates of 83.58% and 89.51%, respectively (Figure 1D). The hydroxyl free radical scavenging potential of GSEA is weaker than other activities, showing a dose-dependent linear increase (R^2^ = 0.995), reaching a peak scavenging activity of 67.13% at a concentration of 5 mg/mL (Figure 1E).

To further evaluate the anti-inflammatory effects of GSEA *in vivo*, mouse models with ear and paw swelling were used (Figures 1F,G). In the xylene-induced mouse ear swelling model, ASP significantly reduced edema compared with the Mo group (*P* < 0.05). In addition, both the MG and HG groups showed a marked decrease in ear edema (*P* < 0.05 and *P* < 0.01, respectively; Figure 1H). Compared to the Mo group, the ASP, MG, and HG groups showed significantly reduced paw swelling in the ovalbumin-induced paw edema model (Figure 1I). The LG group exhibited inhibition rates of 25.81% for ear edema and 11.94% for paw edema, although not statistically significant compared with the Mo group.

### 3.2 Chemical constituents and blood-absorbed ingredients of GSEA

**FIGURE 2:**
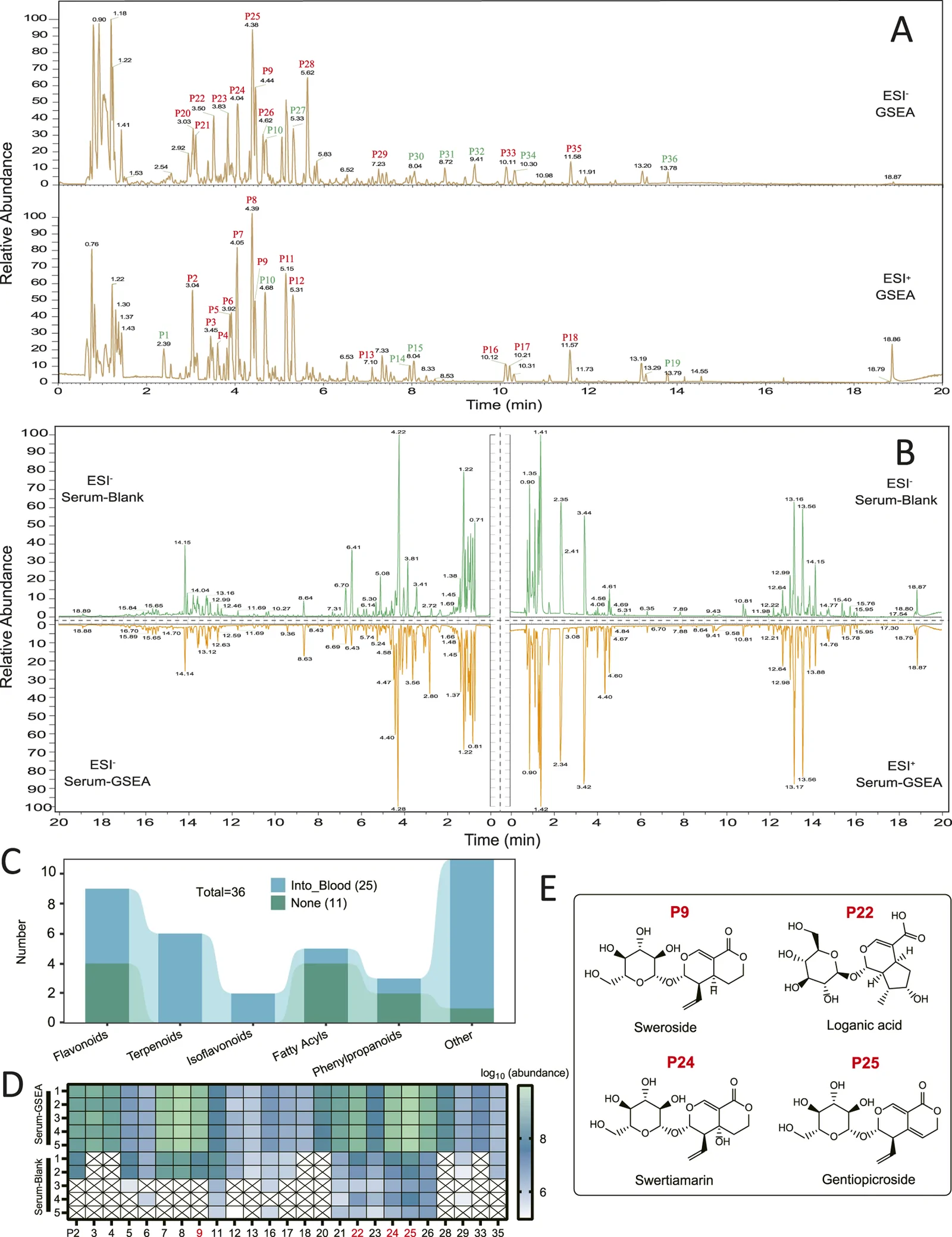
Serum pharmacochemical analysis of GSEA. **(A)** Total ion chromatogram (TIC) of the GSEA extract. **(B)** Comparison of TIC profiles between serum-blank and serum-GSEA. **(C)** Classification of 36 constituents identified in GSEA, including nine flavonoids, six terpenoids, two isoflavonoids, five fatty acyls, three phenylpropanoids, and 11 additional compounds, along with their absorption status: 25 blood-absorbed and 11 non-absorbed compounds. **(D)** Relative abundance profiling of the 25 blood-absorbed ingredients. **(E)** Four key candidate bioactive constituents were identified in the systemic circulation following GSEA administration.

The chemical constituents (Figure 2A) and blood-absorbed ingredients of GSEA after oral administration in mice (Figure 2B) were identified using UHPLC-HRMS. The collected data were analyzed using MS/MS spectral library searches, identifying compounds through mass accuracy and spectral matching scores. Altogether, 36 compounds with significant response intensities were effectively identified in GSEA, including nine flavonoids, six terpenoids, two isoflavonoids, five fatty acyls, three phenylpropanoids, and 11 additional compounds (Table 1). *In vivo* analysis verified that 25 compounds were blood-absorbed, with 56% (5/9) being flavonoids, 100% terpenoids (6/6), 100% isoflavonoids (2/2), 20% fatty acyls (1/5), 33% (1/3) phenylpropanoids, and 91% (10/11) other compounds (Figure 2C). Figure 2D shows a comparison of the levels of the 25 blood-absorbed ingredients in Serum-Blank and Serum-GSEA, with sweroside (P9), loganic acid (P22), swertiamarin (P24), and gentiopicroside (P25) being the main blood-absorbed ingredients. Our previous research reveals that these compounds are terpenoids and key candidate bioactive constituents of *Gentiana* species, and Figure 2E illustrates their chemical structures.

**TABLE 1:** Characterization of chemical compounds of GSEA.

Peak	RT/min	Compound name	m/z	ppm	Adduct	PubCHEM CID	Categories	Status
P1	2.43	Phenylalanine	166.09	17.6	[M+H]^+^	6140	Other	None
P2	3.06	3′,4′-methylenedioxy-N-tert-butylcathinone	194.08	0.5	[M+H−C_4_H_8_] ^+^	85710946	Other	Into_Blood
P3	3.46	Methylferulic acid	167.07	0.1	[M+H−C_2_H_2_O]^+^	717531	Phenylpropanoids	Into_Blood
P4	3.62	Azelaic acid	171.10	0.5	[M+H−H_2_O]^+^	2266	Fatty acyls	Into_Blood
P5	3.89	2″-o-beta-l-galactopyranosylorientin	611.16	0.8	[M+H]^+^	44257952	Flavonoids	Into_Blood
P6	3.95	Tebuconazol	308.15	11.4	[M+H]^+^	86102	Other	Into_Blood
P7	4.02	bk-MDDMA	177.05	0.5	[M+H−C_2_H_7_N]^+^	9794472	Other	Into_Blood
P8	4.39	Gibepyrone D	195.07	0.2	[M+H]^+^	20056005	Terpenoids	Into_Blood
P9	4.45	Sweroside	197.08	0.2	[M+H−C_6_H_10_O_5_]^+^	161036	Terpenoids	Into_Blood
P10	4.69	Isoorientin	449.11	0.6	[M+H]^+^	114776	Flavonoids	None
P11	5.16	Isovitexin	433.11	0.7	[M+H]^+^	162350	Flavonoids	Into_Blood
P12	5.32	7,10-dihydroxy-9,14-dimethoxy-2-methyl-3,4,5,6-tetrahydro-2H,8H-2,6-epoxyoxocino[3,2-b]xanthen-8-one	343.08	0.7	[M+H−C_3_H_6_O]^+^	24039292	Other	Into_Blood
P13	7.11	Wistin	461.14	0.7	[M+H]^+^	10095770	Isoflavonoids	Into_Blood
P14	7.95	4-(3,4-dihydroxyphenyl)-7-methoxy-2-oxo-2H-chromen-5-yl 6-O-beta-D-xylopyranosyl-beta-D-glucopyranoside	463.12	0.9	[M+H−C_5_H_8_O_4_]^+^	14134097	Flavonoids	None
P15	8.04	2′,4,4′,6′-tetramethylether-3′-prenylchalcone	438.24	23.5	[M+ACN+H]^+^	13941333	Phenylpropanoids	None
P16	10.12	Formononetin	269.08	0.2	[M+H]^+^	5280378	Isoflavonoids	Into_Blood
P17	10.21	5,7-dimethoxyluteolinidin cation	299.09	1	[Cat]^+^	102124988	Flavonoids	Into_Blood
P18	11.57	Clobazam	301.07	11.9	[M+H]^+^	2789	Other	Into_Blood
P19	13.78	(9Z,12Z,15Z)-octadecatrienoic acid	279.23	1.7	[M+H]^+^	5280934	Fatty acyls	None
P20	3.02	Violaceol I	139.04	8.4	[M−H−C_7_H_6_O_2_^−^	100615	Other	Into_Blood
P21	3.09	Benzoic acid + 2O, O-hex	315.07	5.7	[M−H]^−^	76211590	Other	Into_Blood
P22	3.51	Loganic acid	375.13	1.2	[M−H]^−^	89640	Terpenoids	Into_Blood
P23	3.84	Secologanoside	389.11	1.6	[M−H]^−^	14136854	Terpenoids	Into_Blood
P24	4.07	Swertiamarin	419.12	1.3	[M+HCOO] ^−^	442435	Terpenoids	Into_Blood
P25	4.4	Gentiopicroside	401.11	2	[M+FA−H]^−^	88708	Terpenoids	Into_Blood
P26	4.62	4,5-dihydroxyphthalic acid	153.02	6.8	[M−H−CO_2_]^−^	45746	Other	Into_Blood
P27	5.33	Scoparin	461.11	5.8	[M−H]^−^	20055255	Flavonoids	None
P28	5.62	Robustaside D	179.03	5.2	[M−H−C_12_H_14_O_6_]^−^	38358972	Other	Into_Blood
P29	7.25	(5-benzoyloxy-1,2,6-trihydroxycyclohex-3-en-1-yl) methyl benzoate	419.10	17.7	[M+Cl]^−^	14283260	Other	Into_Blood
P30	8.05	Cumaroylspermidine	582.26	1.3	[M−H]^−^	14777879	Phenylpropanoids	None
P31	8.73	FA 18:2 + 3O	327.22	2.9	[M-H]-	72732143	Fatty acyls	None
P32	9.4	FA 18:1 + 3O	329.23	5.1	[M−H]^−^	153001	Fatty acyls	None
P33	10.12	7-hydroxy-3′-methoxyflavone	267.07	1.9	[M−H]^−^	5393153	Flavonoids	Into_Blood
P34	10.31	Luteolin 7-methyl ether	299.06	0	[M−H]^−^	5318214	Flavonoids	None
P35	11.58	5-hydroxy-2-(4-hydroxyphenyl)-7-methoxy-4-oxo-4H-chromen-3-yl 2-O-beta-D-galactopyranosyl-beta-D-glucopyranoside	299.06	2.1	[M−H−C_12_H_20_O_10_]^−^	45359931	Flavonoids	Into_Blood
P36	13.78	FA 18:2+1O	295.23	0.4	[M−H]^−^	1,424	Fatty acyls	None

### 3.3 Network pharmacology analysis of GSEA against NAFLD

**FIGURE 3:**
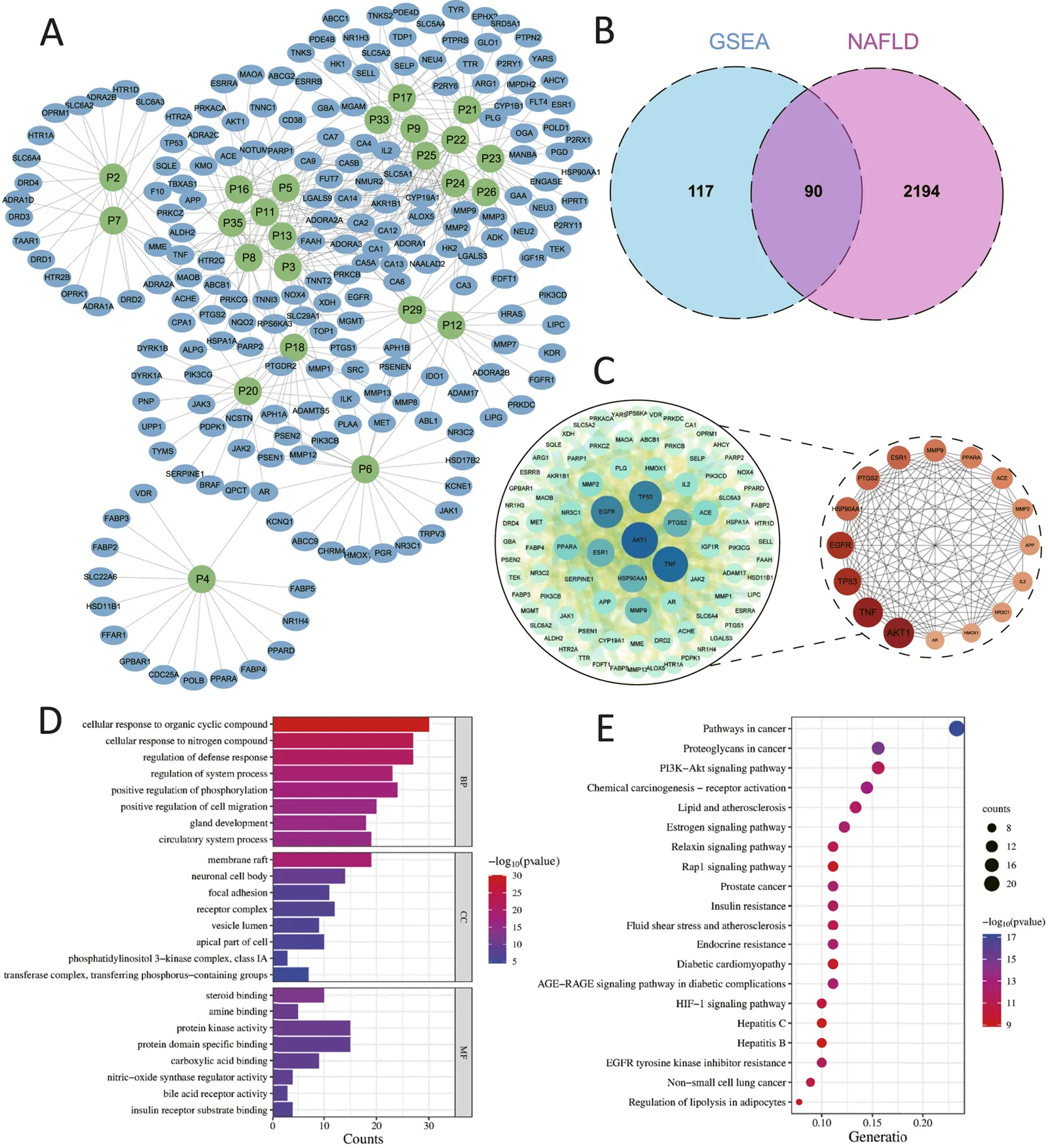
Analysis of Network Pharmacology. **(A)** A network diagram showing the interactions between blood-absorbed ingredients and their predicted targets. **(B)** Venn diagram showing the common targets. **(C)** The protein-protein interaction (PPI) network was constructed from the common targets. **(D,E)** GO and KEGG enrichment analyses.

**FIGURE 4:**
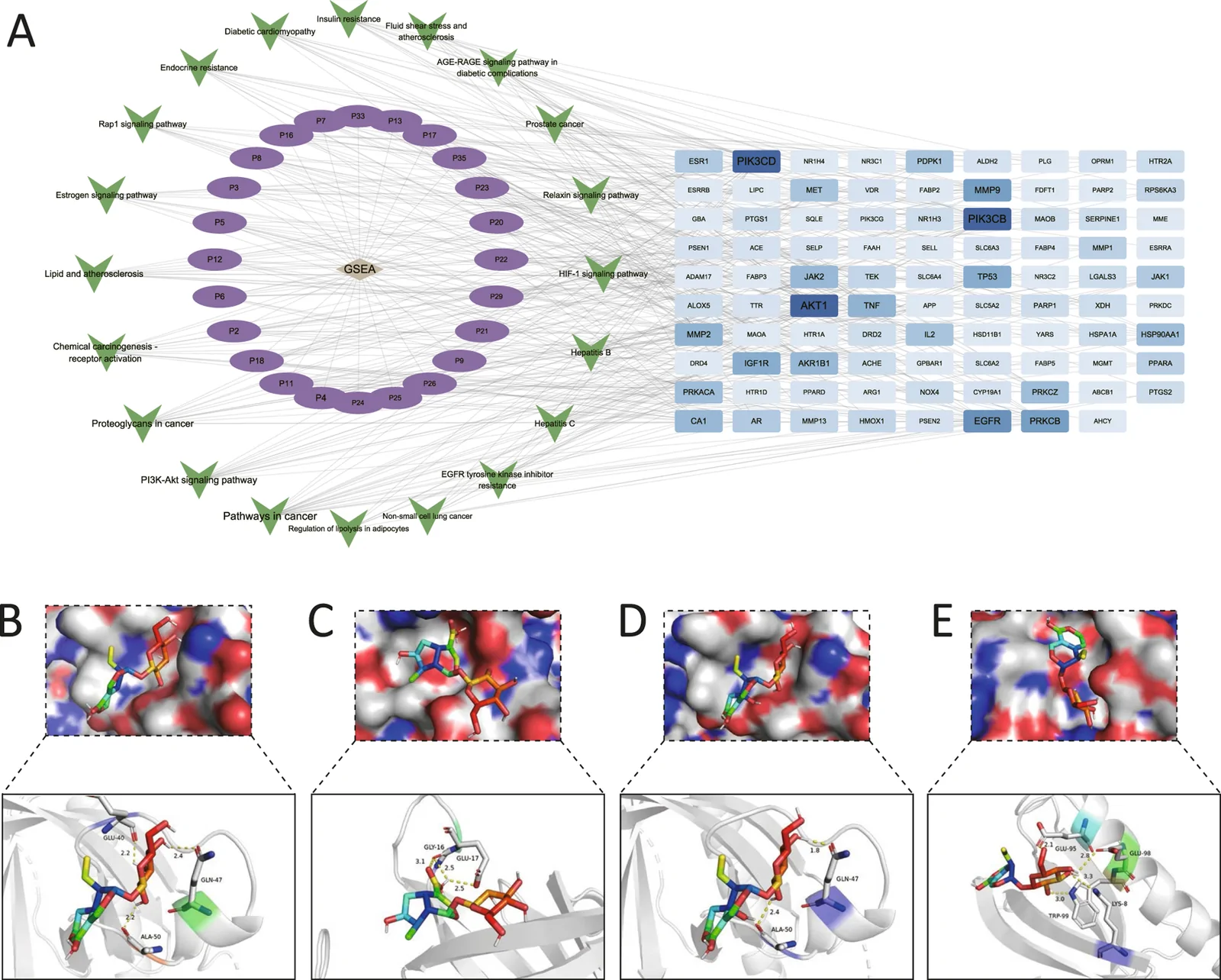
Network construction of compound-target-pathway and analysis of molecular docking. **(A)** An integrated network displaying the interactions among key candidate bioactive compounds, their molecular targets, and associated signaling pathways. **(B–E)** Molecular docking of sweroside, loganic acid, swertiamarin, and gentiopicroside with AKT1.

Using UHPLC-HRMS, 25 blood-absorbed ingredients of GSEA were identified, leading to the prediction of 207 potential therapeutic targets (Figure 3A). By intersecting these with known NAFLD targets, 90 common targets were identified (Figure 3B). Analysis of the PPI network showed that the 90 targets resulted in 592 interaction pairs, with 89 interconnected nodes and 1 node remaining isolated. Key hub proteins with high degrees of connectivity included AKT1, TNF, and TP53 (Figure 3C). Functional enrichment analysis indicated that these targets were predominantly located in membrane rafts, involved in biological processes such as cellular response to organic cyclic compounds, and were primarily linked to the molecular functions of steroid binding (Figure 3D). Among the 157 identified KEGG pathways, the top 20 significantly enriched pathways included “Pathways in cancer,” the “PI3K-Akt signaling pathway,” and “Regulation of lipolysis in adipocytes” (Figure 3E). An extensive visualization of the compound-target-pathway network emphasized key critical nodes predominantly associated with PIK3CB, PIK3CD, and AKT1 (Figure 4A). Recognizing AKT1 as a critical target in both the PPI and compound-target-pathway networks, we performed molecular docking to predict its binding affinities with four key candidate bioactive compounds: sweroside, loganic acid, swertiamarin, and gentiopicroside. Our findings showed that each of the four AKT1-compound complexes had predicted binding energies below −5 kcal/mol. Loganic acid demonstrated a significant interaction with a binding energy of −6.2 kcal/mol, and sweroside exhibited a similar interaction at −5.9 kcal/mol. With binding energies of −6.0 and −6.1 kcal/mol, swertiamarin and gentiopicroside displayed moderate binding affinities, respectively. The binding modes, mainly stabilized by hydrogen bonds, are illustrated in Figures 4B–E.

### 3.4 GSEA reduces liver damage and fat accumulation in mice

**FIGURE 5:**
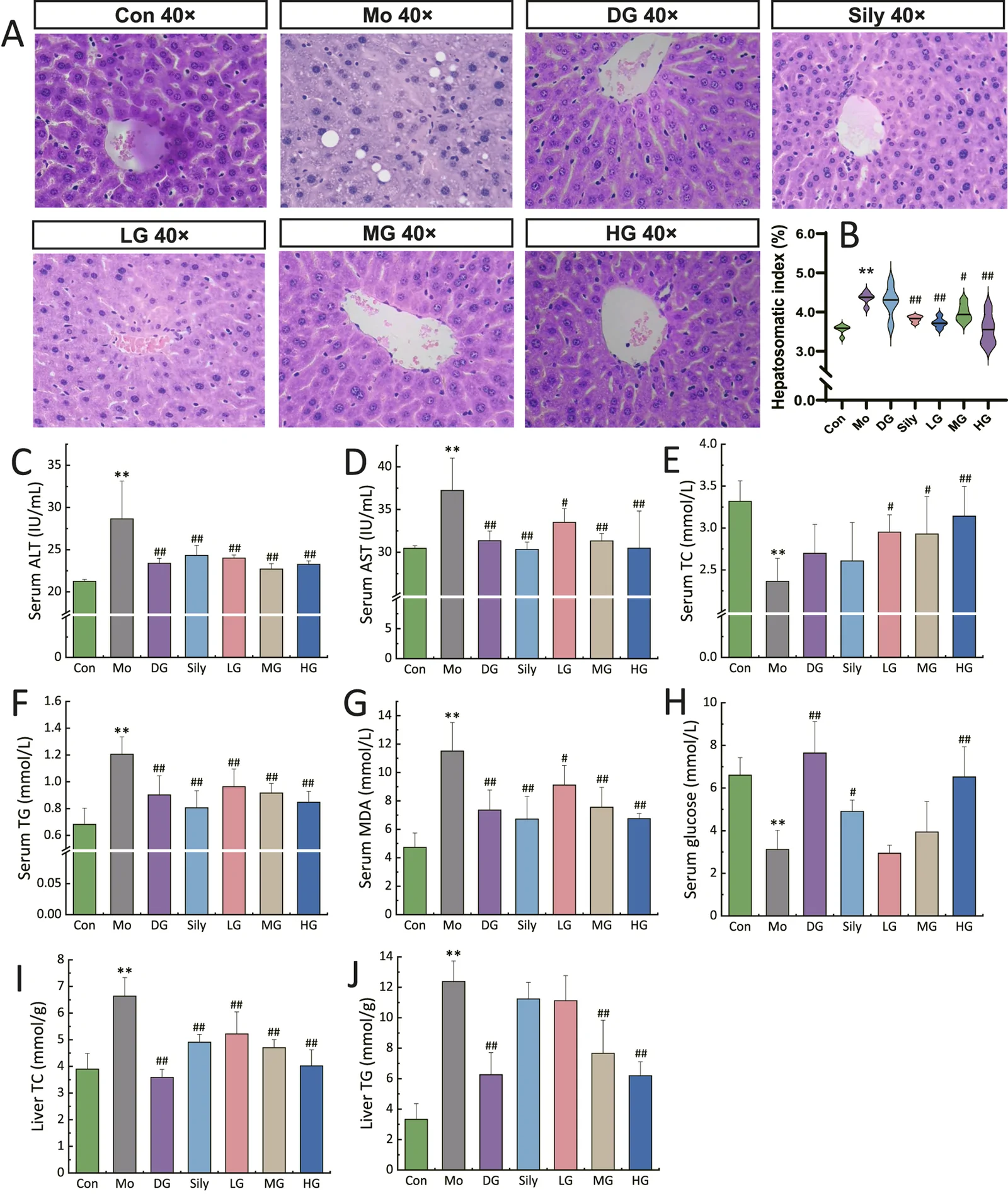
GSEA reduces liver damage and fat accumulation in mice. **(A)** H&E staining of liver tissues. **(B)** Hepatosomatic index. **(C–H)** Levels of serum biochemical markers such as ALT, AST, TC, TG, MDA, and glucose. **(I,J)** Levels of TC and TG in the liver. Data are presented as mean ± SEM (n = 5). ^**^ *P* < 0.01 vs. Con group; ^#^ *P* < 0.05, ^##^ *P* < 0.01 vs. Mo group.

H&E staining indicated that the Con group had normal liver cells and maintained structural integrity. Conversely, the liver in the Mo group exhibited remarkable pathological changes, including cytoplasmic vacuolization, disruption of cellular structures, and disarray of the hepatic cords. After pharmacological treatment, all treated groups exhibited varying degrees of histological improvement except for the LG group, which showed minimal nonsignificant changes. The MG and HG groups showed marked improvements (Figure 5A). Additionally, the hepatosomatic index (liver-to-body weight ratio) in the Mo group was markedly higher than that of the Con group (*P* < 0.01). Compared to the Mo group, all treatment groups except for the DG group showed a significant reduction in the hepatosomatic index (*P* < 0.05) (Figure 5B). The HG group demonstrated the most notable enhancement in liver condition.

Novel promising biomarkers linked to liver injury and lipid metabolism, including serum alanine aminotransferase (ALT), aspartate aminotransferase (AST), total cholesterol (TC), triglycerides (TG), glucose, and malondialdehyde (MDA), were assessed in both serum and liver tissue. As shown in Figures 5C,D, the serum ALT and AST levels in the Mo group were significantly higher than those in the Con group (*P* < 0.01), indicating liver cell damage, which aligns with the histopathological results. The Mo group also showed significantly increased serum TG and MDA levels, together with elevated hepatic TC and TG contents (*P* < 0.01). By contrast, serum TC and glucose levels were significantly lower in the Mo group than in the Con group. Following 2 weeks of treatment with diammonium glycyrrhizinate, silymarin, LG, MG, and HG, all parameters exhibited enhancement compared to the Mo group (Figures 5E–J). The results indicate that diammonium glycyrrhizinate, silymarin, and GSEA mitigated hepatic lipid metabolism. High-dose GSEA showed a promising therapeutic effect among these treatments.

### 3.5 GSEA alleviates inflammatory response and glycolipid metabolism

**FIGURE 6:**
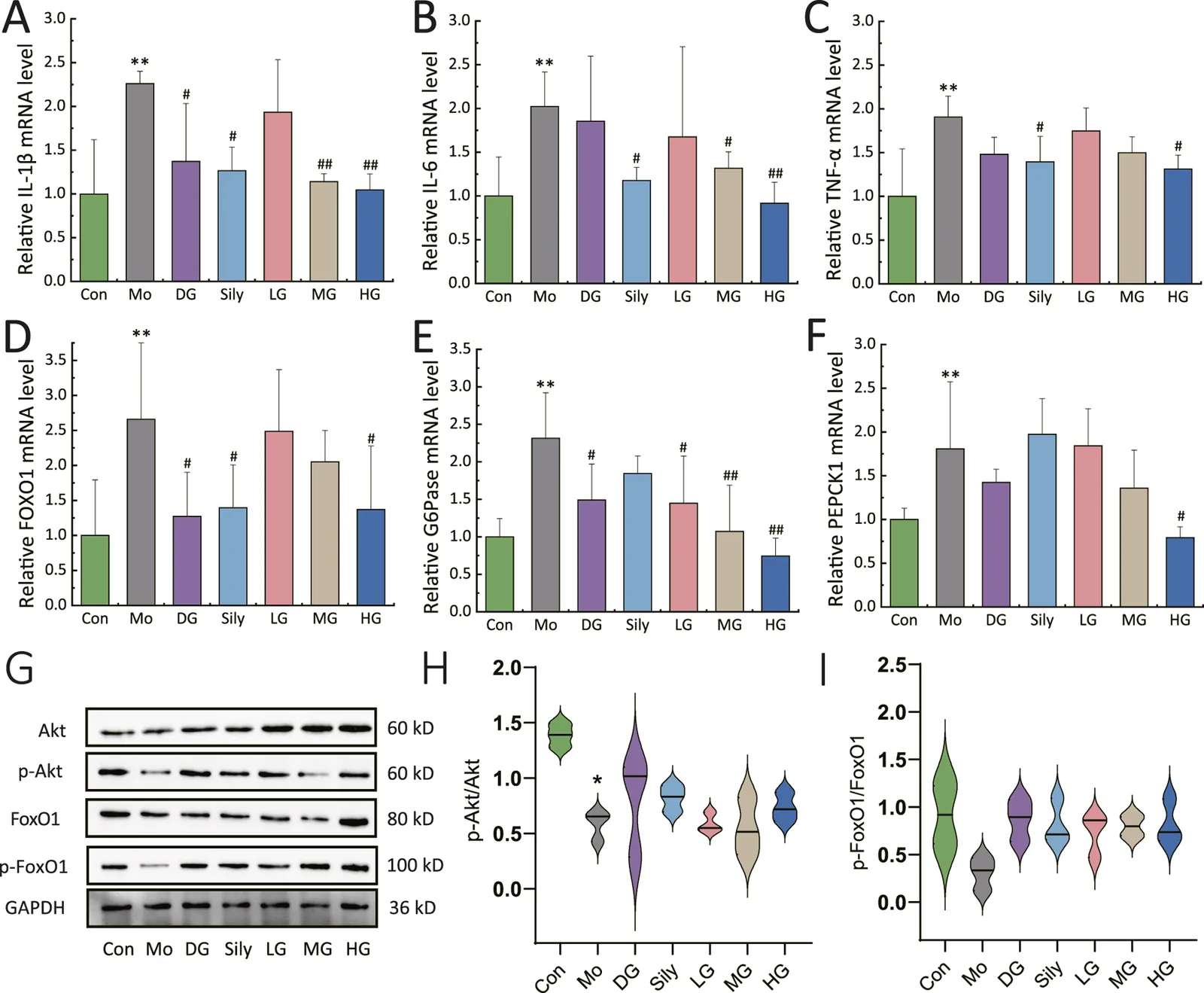
GSEA alleviates inflammation and reestablishes balance in glycolipid metabolism. **(A–C)** Liver mRNA expression of pro-inflammatory cytokines such as IL-1β, IL-6, and TNF-α. **(D–F)** Relative mRNA expression levels of genes associated with glucose and lipid metabolism, including *FOXO1, G6Pase,* and *PEPCK1*. **(G–I)** The ratios of p-Akt/Akt and p-FoxO1/FoxO1. Data are presented as mean ± SEM (n = 5). ^**^ *P* < 0.01 vs. Con group; ^#^ *P* < 0.05, ^##^ *P* < 0.01 vs. Mo group.

Figures 6A–F show that the Mo group had significantly higher hepatic mRNA expression levels of inflammatory cytokines (IL-1β, IL-6, and TNF-α) and glycolipid metabolism-related genes (*FOXO1*, *G6Pase*, and *PEPCK1*) than the Con group (*P* < 0.01). Following 2 weeks of treatment with DG, Sily, or different doses of GSEA, the expression levels of these genes were reduced compared to the Mo group, with pronounced effects in the HG group. The results suggest that GSEA successfully lessens inflammation and glycolipid metabolic issues linked with liver injury induced by tetracycline and HFD.

Numerous studies have demonstrated the essential regulatory functions of the Akt/FoxO1 signaling pathway in hepatic regeneration, and various approaches to treating liver diseases focus on understanding the molecular mechanisms of liver injury and recovery. This research showed that network pharmacology highlighted multiple targets enriched in the PI3K/Akt signaling pathway and also implicated FoxO1-related signaling, although the degree of enrichment was less pronounced. Consequently, Western blot analysis showed that the hepatic p-Akt/Akt ratio was significantly lower in the Mo group than in the Con group (*P* < 0.05); in contrast, the difference in the p-FoxO1/FoxO1 ratio was not statistically significant. Although numerical changes were observed after treatment, neither ratio differed significantly between the GSEA-treated and Mo groups (Figures 6G–I). Therefore, these findings provide preliminary associative evidence that GSEA acts through the PI3K/Akt/FoxO1 signaling pathway.

### 3.6 GSEA improves the balance in gut microbiota

**FIGURE 7:**
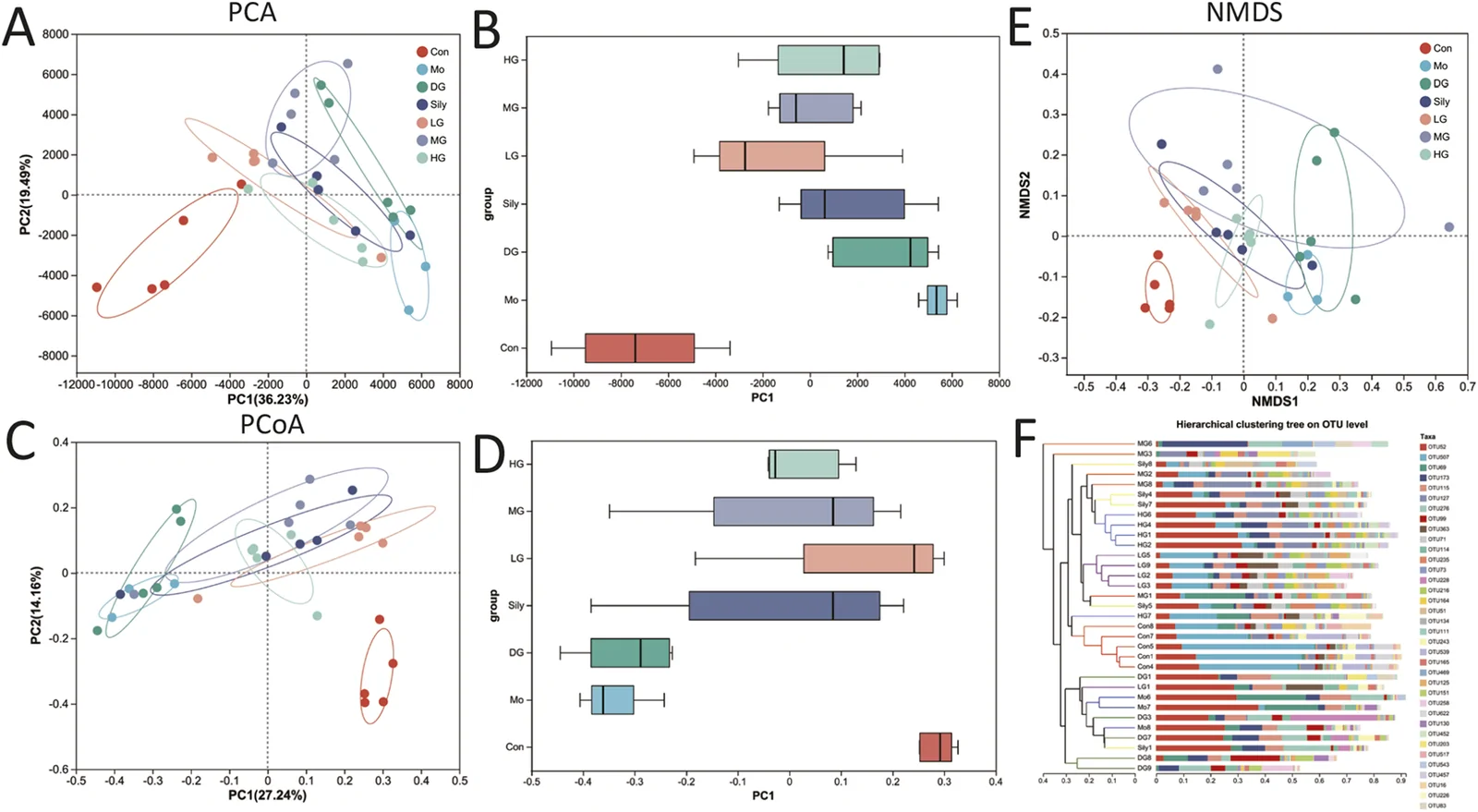
GSEA influences the composition of the gut microbiome in mice. **(A,B)** Principal component analysis (PCA) and related comparisons along principal component 1 (PC1). **(C,D)** Principal coordinates analysis (PCoA) and related comparisons along PC1. **(E)** Non-metric multidimensional scaling (NMDS). **(F)** Phylogenetic tree illustrating microbial evolutionary relationships (n = 5).

According to 16S rDNA sequencing, GSEA improves the gut microbiota composition. The impact of GSEA on gut microbiota variations across all experimental groups was clarified using Principal Component Analysis (PCA), Principal Coordinates Analysis (PCoA), and Non-Metric Multidimensional Scaling (NMDS), with the Mo group being distinct (Figures 7A–E). The HG, LG, and Sily groups showed similar effects to the Con group following pharmacological treatment, clustering together. The second principal component (PC2) in both PCA and PCoA indicated that the gut microbiome structure in the Mo group recovered to resemble the Con group more closely after treatment, and the LG group exhibited notable convergence (Figures 7B,D). The phylogenetic tree additionally reinforced the previously mentioned observations (Figure 7F).

**FIGURE 8:**
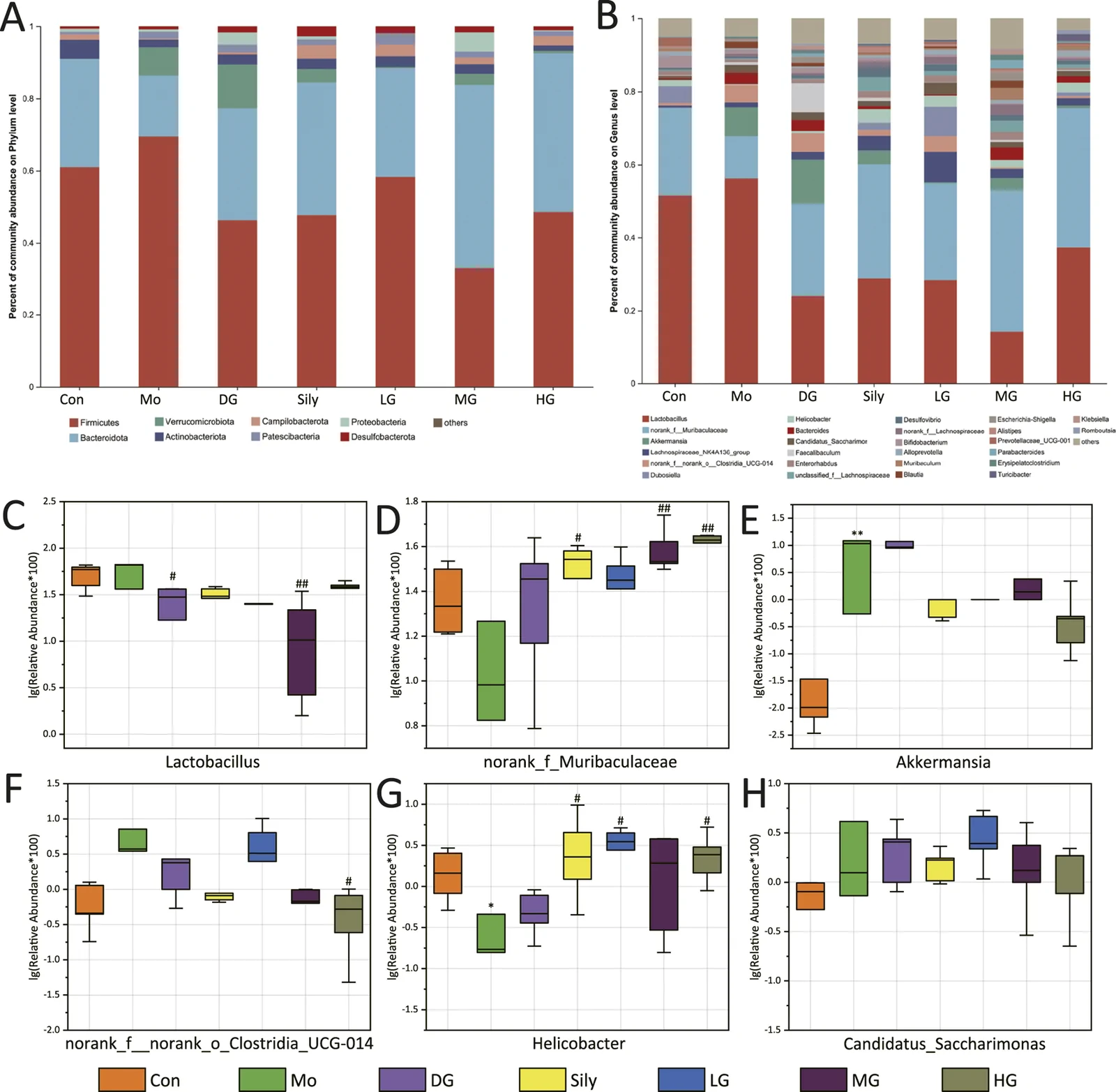
GSEA modifies gut microbes at the phylum and genus levels. **(A,B)** The relative abundance of microbial taxa at the levels of phylum and genus. **(C–H)** Changes in the abundance of dominant genera, such as *Lactobacillus*, *norank_f_Muribaculaceae*, *Akkermansia*, *norank_f_norank_o_Clostridia_UCG-014*, *Helicobacter*, and *Candidatus_Saccharimonas*. Data are presented as mean ± SEM (n = 5). ^*^ *P* < 0.05, ^**^ *P* < 0.01 vs. Con group; ^#^ *P* < 0.05, ^##^ *P* < 0.01 vs. Mo group.

Changes in gut microbiota composition at the phylum level are shown in Figure 8A. Despite variations in relative abundances, Firmicutes and Bacteroidota were consistently the dominant phyla across all groups. The Mo group showed a much higher abundance of Firmicutes and a significantly lower abundance of Bacteroidota compared to the Con group. The alterations were partially reversed to a varying degree after GSEA treatment. Our results revealed a significantly higher Firmicutes/Bacteroidota ratio in the Mo group, suggesting an imbalance in gut microbiota. The imbalance in microbial composition was effectively reversed after GSEA administration, with the LG group showing a microbial profile similar to that of the control group. Figure 8B illustrates the relative abundance at the genus level across different groups. Figures 8C–H emphasize changes in several important microbial taxa. In comparison to the Con group, the Mo group showed a decrease in the relative abundances of *norank_f_Muribaculaceae* and *Helicobacter*, while *Lactobacillus*, *Akkermansia*, *norank_f_norank_o_Clostridia_UCG-014*, and *Candidatus_Saccharimonas* increased and were more abundant. The treatment with GSEA and Sily partially reversed these changes.

**FIGURE 9:**
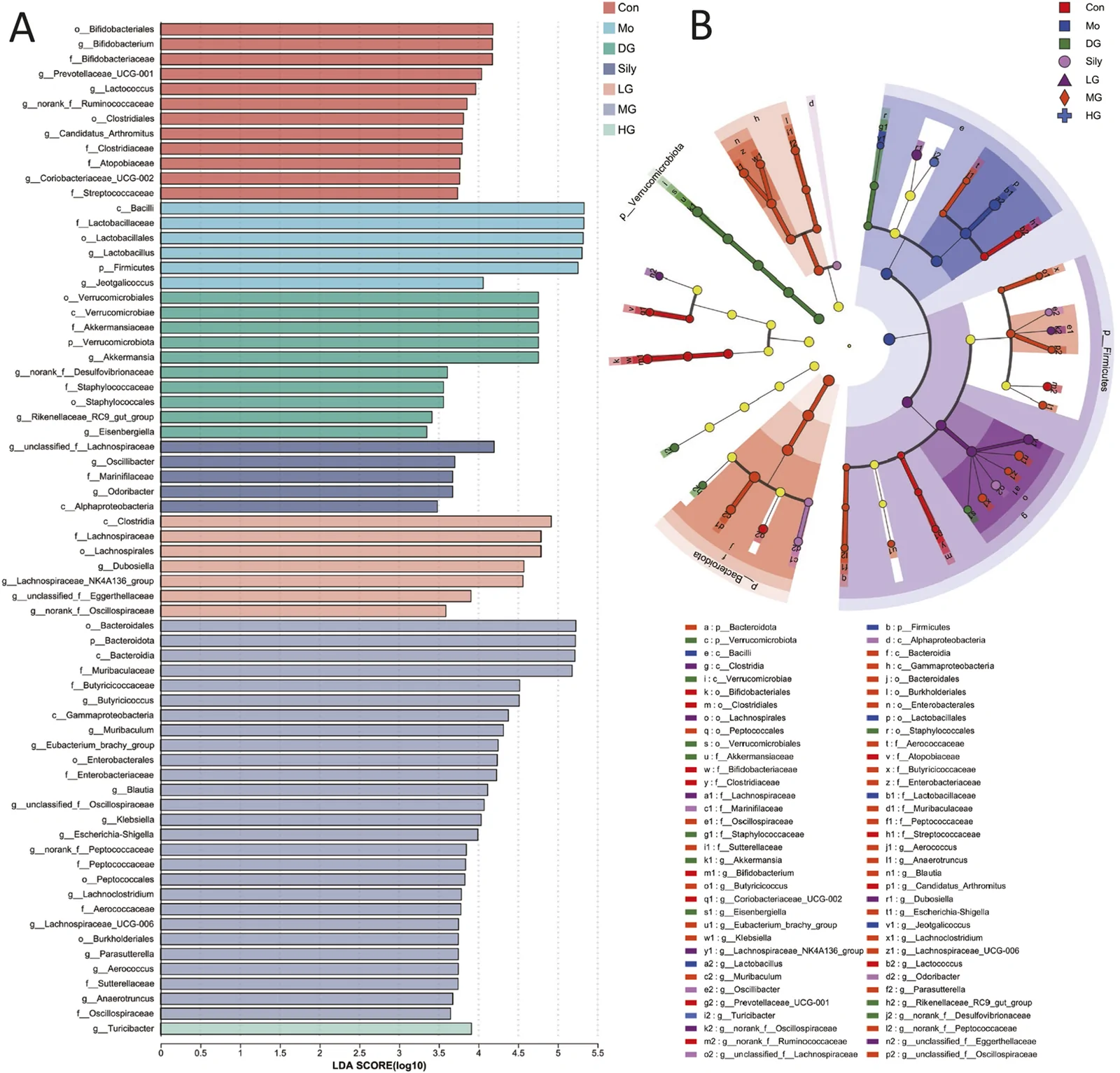
Effect of GSEA on microbial biomarkers by LEfSe analysis. **(A)** Bar chart of LDA scores illustrating taxa with differential abundance. **(B)** Cladogram depicting the phylogenetic distribution of biomarker taxa (n = 5).

LEfSe analysis revealed unique gut microbiota biomarkers in the different experimental groups. Using linear discriminant analysis (LDA), biomarkers specific to each group were screened (Figure 9A) and plotted on an LEfSe cladogram, with taxonomic levels represented by rings with phyla in the outermost and genera in the innermost (Figure 9B). At the genus level, the Con group was marked by an abundance of *Bifidobacterium* and *Prevotellaceae_UCG-001*. *Lactobacillus* and *Jeotgalicoccus* were characterized in the Mo group. The DG group was associated with *Akkermansia* and *Eisenbergiella*, the Sily group with *Oscillibacter* and *Dooribacter*, and the LG group with *Dubosiella*. The MG group showed the highest variety of distinct biomarkers, including *Butyricicoccus*, *Muribaculum*, *Blautia*, *Lachnoclostridium*, *Lachnospiraceae_UCG-006*, *Parasutterella*, *Aerococcus*, and *Anaerotruncus*, while the HG group had only one distinctive biomarker, *Turicibacter*. These findings demonstrate distinct alterations in gut microbial biomarkers and suggest that GSEA may alleviate gut microbiota dysbiosis associated with HFD- and tetracycline-induced liver injury.

## 4 Discussion

The rising occurrence of NAFLD, driven by unhealthy dietary habits and physical inactivity, has made the chronic liver condition a major and increasing public health challenge. However, Pharmacological options for NAFLD remain limited and are restricted to selected disease stages. Inappropriate medications under NAFLD conditions, including prolonged administration of anti-tuberculosis drugs in humans and the inclusion of antibiotics in animals to prevent infections and promote growth, may further aggravate liver injury (Shen et al., 2019; Hou et al., 2024). It is crucial to advance high-quality new therapeutic agents or functional foods to tackle and alleviate this type of simultaneous liver injury.

The genus *Gentiana* is known for its high levels of terpenoids, including swertiamarin and gentiopicroside, which possess a wide range of potent pharmacological activities, confirming their potential as a promising option for pharmacotherapy. Despite numerous reports on the antioxidant properties of *Gentiana* extracts, the exceptional antioxidant activity may be correlated to swertiamarin or other active phytochemicals rather than gentiopicroside (Wani et al., 2013; Azman et al., 2014; Wang et al., 2024). This research is the first to systematically assess the pharmacological effects of GSEA, including its antioxidant, anti-inflammatory, and hepatoprotective properties. *In vitro* antioxidant assays revealed its significant ability to neutralize ABTS, DPPH, and hydroxyl radicals. *In vivo* evaluations also demonstrated that GSEA notably decreased xylene-induced ear swelling and ovalbumin-induced paw swelling in mice, aligning with previous reports on the anti-inflammatory properties of gentiopicroside and swertiamarin (Chang et al., 2021; Wang et al., 2024; Wu et al., 2017; Xu et al., 2021). Collectively, these results suggest that GSEA exerts strong antioxidant and anti-inflammatory properties, emphasizing its potential for therapy and warranting further investigation into the mechanism.

Conventional network pharmacology methods, which rely on database mining or bioactive identification from literature, are naturally restricted in chemical completeness and biological significance. In this study, we applied serum pharmacochemistry to pinpoint and confirm the blood-absorbed ingredients of GSEA both *in vitro* and *in vivo* settings. A total of 36 compounds were identified in GS extract, of which 25 were later found in Serum-GSEA. Among these, sweroside, loganic acid, swertiamarin, and gentiopicroside are key components of the iridoid subclass of terpenoids, which were detected at significantly elevated levels in the systemic circulation. Earlier studies have identified these compounds as key phytochemicals in *Gentiana* species and noted their common presence in raw forms (Gibitz-Eisath et al., 2022). The documented hepatoprotective, anti-inflammatory, and antioxidant effects of swertiamarin and gentiopicroside are complemented by the elevated serum levels of sweroside and loganic acid, indicating their potential pharmacological activity in GSEA. Sweroside demonstrates anti-inflammatory effects by inhibiting the NF-κB signaling pathway and enhancing SIRT1 expression (Wang et al., 2021) and has the potential to ameliorate NAFLD by regulating lipid metabolism and inflammatory responses (Yang G. et al., 2020; Yang Q. L. et al., 2020). Similarly, loganic acid shows antioxidant and anti-inflammatory effects through the TLR4/NF-κB pathway and the SIRT1/Nrf2 signaling axis (Prakash et al., 2023). Network pharmacology analysis revealed 90 common target genes, highlighting AKT1, TNF, and TP53 as major hub nodes in the PPI network. AKT1 is crucial for controlling essential cellular activities such as survival, proliferation, and metabolism. TNF acts as a key proinflammatory cytokine, while TP53 is a classic tumor suppressor involved in cancer development. Research has proved that HFD can encourage liver cancer development *via* the Akt signaling pathway (Bu et al., 2024; Ren et al., 2024). KEGG enrichment analysis revealed that these targets are linked to cancer, the PI3K/Akt signaling pathway, and the regulation of lipolysis in fat cells. The molecular docking analysis indicated that the core compounds of GSEA have strong binding affinities with AKT1, implying that GSEA modulates this crucial signaling axis.

Earlier research has indicated that NAFLD and DILI are pathologically marked by increased oxidative stress, disrupted mitochondrial *β*-oxidation, and drug-induced increases in hepatic fatty acid uptake (Rodriguez-Diaz et al., 2022). NAFLD can make the liver more susceptible to drug-induced liver toxicity, triggering or worsening the condition (Fromenty, 2013; Lammert et al., 2019). Hepatic lipid buildup results in a metabolic imbalance, increased fatty acid intake, and new fat production that exceeds *β*-oxidation and triglyceride export, aggravating inflammatory responses in the liver (Ipsen et al., 2018; Liu et al., 2024). Approaches to managing fatty liver diseases are essential for managing NAFLD and DILI effectively. In this study, mice in the Mo group showed a higher hepatosomatic index and significant accumulation of lipid droplets in liver cells. Administering different substances, such as GSEA and sily, resulted in significant decreases in the hepatosomatic index and liver lipid content, consistent with biochemical evaluations of TC and TG in liver tissue. Elevated serum ALT and AST indicated liver damage, including vacuolization, cell disruption, and disorganized hepatic cords. Interestingly, GSEA led to reduced MDA levels, a well-known marker of oxidative damage, highlighting its potent antioxidant properties.

NAFLD is frequently linked to insulin resistance, with the PI3K/Akt signaling pathway being activated by insulin. Research indicated that this pathway is dynamically regulated as the disease progresses (Li et al., 2021). The activation of PI3K triggers phosphorylation cascades, leading to the activation of Akt and the phosphorylation of downstream targets involved in cell growth, metabolism, and survival. FoxO1, a key transcription factor, is tightly regulated *via* the PI3K/Akt signaling axis. In conditions like NAFLD, which are insulin-resistant, FOXO1 gathers in the nucleus. It stimulates gluconeogenesis by increasing the expression of genes such as PEPCK and G6Pase, promoting hepatic glucose production (Ge et al., 2021). Our research revealed that GSEA notably increased hepatic p-Akt expression and promoted FoxO1 phosphorylation, consistent with previous studies showing that gentiopicroside and sweroside can ameliorate glucolipid metabolic disorders *via* the PI3K/Akt signaling pathway (Xiao et al., 2022; Ma et al., 2024).

The liver serves as the main metabolic hub for substances absorbed through the intestinal epithelium, highlighting a direct connection between gut microbiota and liver metabolic activities. Recent research indicates that microbial metabolites can lead to the accumulation of microbial-derived substrates, which in turn may trigger toxic effects in the liver (Niu and Chen, 2020). In individuals with NAFLD and DILI, diet changes and intensive antibiotic use can disrupt gut microbiota, resulting in altered microbial metabolite profiles. After being absorbed into the bloodstream, these metabolites travel to the liver, where they engage with hepatic signaling pathways and enzymes that metabolize foreign substances, possibly influencing drug pharmacokinetics and leading to liver toxicity. The gut microbiota of the Mo group mice showed significant changes, with greater taxonomic differences and less similarity compared to the Con group. Moreover, the Mo group showed a higher Firmicutes/Bacteroidota ratio, suggesting gut microbial imbalance, aligning with previous observations in the NAFLD mouse model (Zhang et al., 2024). At the genus level, the relative abundance of *norank_f_Muribaculaceae* and *Helicobacter* decreased in the Mo group, while *Akkermansia* increased. The genus Muribaculaceae, prominent in the Bacteroidetes phylum in rodent hosts, is vital for fermenting dietary fiber and mucin, thereby producing short-chain fatty acids (SCFAs) such as propionate (Zhu et al., 2024). These SCFAs maintain gut barrier integrity and regulate the immunological response. A decrease in Muribaculaceae in the Mo group might correlate with disease progression. The reduction in *Helicobacter* and the rise in Akkermansia in the Mo group were unexpected, given that *Helicobacter* species are recognized as pathogens related to gastritis, gastric ulcers, and gastric cancer. Generally regarded as a beneficial bacterium, Akkermansia aids in maintaining intestinal barrier integrity, modulating immune responses, and metabolic health (Panzetta and Valdivia, 2024). This unusual finding might be associated with the combined effects of HFD and tetracycline exposure. Tetracycline, a broad-spectrum antibiotic, can inhibit microorganisms such as *Helicobacter*, probably causing microbial imbalance or the overgrowth of non-target microbes. In addition, unique microbial biomarkers associated with the therapeutic response were identified through LEfSe analysis. Importantly, *Oscillibacter* is a prominent bacterium in the Sily group, while *Butyricicoccus, Blautia, Lachnoclostridium*, and *Parasutterella* are significant microorganisms in the MG group. Previous findings have shown that individuals with various *Oscillibacter* species tend to have lower cholesterol levels compared to those lacking these bacteria. This is likely because *Oscillibacter* can break down cholesterol in the gut, decreasing cholesterol levels and reducing the risk of heart disease (Li et al., 2024). Following Sily treatment, the significant increase of this bacterium greatly boosts lipid metabolism in NAFLD. *Butyricicoccus* and *Blautia*, distinctive bacteria in the MG group, produce SCFAs such as butyrate, contributing to gut homeostasis and modifying immune responses (Eeckhaut et al., 2013; Ozato et al., 2019; Mao et al., 2024). Research has demonstrated a notable correlation between *Blautia* and visceral fat area, where *Blautia* encourages the accumulation of visceral fat (Ozato et al., 2019). In a cross-sectional study, mice were given *Blautia* species to reduce obesity and diabetes, altering metabolic pathways and enhancing anti-inflammatory responses (Hosomi et al., 2022). *Lachnoclostridium* improves gut permeability and reduces inflammatory responses, making its increased presence crucial for preventing NAFLD (Dai et al., 2023). Conversely, having fewer *Parasutterella* in the MG group is advantageous for disease management, as elevated levels of this bacterium correlate with higher body mass index and type 2 diabetes (Henneke et al., 2022; Wen et al., 2024). The dysbiosis in the Mo group was partially reversed by GSEA treatment, possibly due to direct interactions between poorly absorbed compounds and the gut microbiota (Feng et al., 2019). Isoorientin, identified in this study as a flavonoid compound, does not enter systemic circulation and is known for its pharmacological properties, including antioxidant and anti-inflammatory activities. Previous research demonstrated that isoorientin alters the gut microbiota, reducing pathogenic bacteria and promoting the growth of existing beneficial bacteria (Yuan et al., 2018; Zhou et al., 2022; Zhou et al., 2025). These findings suggest that Traditional Chinese Medicine could alleviate gut dysbiosis, but the exact roles of many microbial taxa in disease progression are poorly characterized, highlighting the need for additional mechanistic studies such as fecal microbiota transplantation.

To date, studies on NAFLD and DILI have mainly focused on lipid accumulation, persistent inflammation, oxidative stress, gut microbiome imbalance, and impaired signaling pathways. In our study, we demonstrate that the GSEA contains a variety of bioactive substances, particularly terpenoids such as sweroside, loganic acid, swertiamarin, and gentiopicroside. These components show significant anti-inflammatory and antioxidant properties. UHPLC-HRMS was used to characterize 25 bioavailable constituents in serum-GSEA, and network pharmacology was applied to predict their potential targets, regulatory mechanisms, and therapeutic significance in the context of NAFLD-DILI comorbidity. Following this, we comprehensively analyzed the multifaceted therapeutic effects and potential mechanisms of GSEA, emphasizing its influence on lipid metabolism, inflammation, oxidative stress, gut microbiota, and the activation of the PI3K/Akt/FoxO1 pathway in liver injury caused by the combination of an HFD and tetracycline exposure. Notably, GSEA components that are bioavailable but poorly absorbed into the bloodstream can directly interact with the gut microbiota, influencing the ecological balance of intestinal microbes and aiding in gut restoration. This comprehensive pharmacological study offers an organized insight into the pharmacological functions of *Gentiana sino-ornata* Balf. f., highlighting its potential as a multi-target therapeutic strategy for treating NAFLD and DILI.

## Data Availability

The original contributions presented in the study are publicly available. The 16S rRNA gene sequencing data can be found in the NCBI Sequence Read Archive (SRA) under BioProject accession number PRJNA1519097.

## Glossary

Akt: protein kinase B
ALT/AST: alanine/aspartate aminotransferase
ASP: aspirin
DG: diammonium glycyrrhizinate
DILI: drug-induced liver injury
GAPDH: glyceraldehyde 3-phosphate dehydrogenase
GO: Gene Ontology
GS: Gentiana sino-ornata
GSEA: GS ethyl acetate extract
H&E: hematoxylin and eosin staining
HG: high-dose group of GSEA
HPLC: high-performance liquid chromatography
KEGG: Kyoto Encyclopedia of Genes and Genomes
LDA: linear discriminant analysis
LG: low-dose group of GSEA
MASLD: metabolic dysfunction-associated steatotic liver disease
MDA: malondialdehyde
MG: medium-dose group of GSEA
Mo: model group
NAFLD: nonalcoholic fatty liver disease
NMDS: non-metric multidimensional scaling
OTUs: operational taxonomic units
PCA: principal component analysis
PCoA: principal coordinates analysis
PPI: protein interaction network
qRT-PCR: quantitative real-time polymerase chain reaction
SCFAs: short-chain fatty acids
SEM: standard error of the mean
Sily: silymarin
TC: total cholesterol
TG: triglycerides
TICs: total ion chromatograms
UHPLC-HRMS: ultra-high-performance liquid chromatography coupled with high-resolution mass spectrometry
VC: vitamin C
