# Supplementary material for: Gentiana sino-ornata Balf. f. ethyl acetate extract alleviates high-fat diet- and tetracycline-induced liver injury in mice: insights from serum pharmacochemistry, network pharmacology, and experimental validation
Source: Front Pharmacol. 2026 Sep 8;17:1923448. doi: 10.3389/fphar.2026.1923448 (PMC13594584; doi:10.3389/fphar.2026.1923448)
Supplement: Supplementary file 1 [file DataSheet1.docx]

***Supplementary materials***

**Table S1. Sequences of primers used for RT-qPCR.**

| Genes | Nucleotide sequence of primers (5′ to 3′) | | Length (bp) |
| --- | --- | --- | --- |
| *IL-1β* | Forward | GCCCATCCTCTGTGACTCAT | 230 |
|  | Reverse | AGGCCACAGGTATTTTGTCG |  |
| *IL-6* | Forward | CTCCCAACAGACCTGTCTATAC | 97 |
|  | Reverse | CCATTGCACAACTCTTTTCTCA |  |
| *TNF-α* | Forward | GGGAGCAAAGGTTCAGTGAT | 128 |
|  | Reverse | CCTGGCCTCTCTACCTTGTT |  |
| *FoxO1* | Forward | GCTCTGTGCGCCTAAGTACA | 251 |
|  | Reverse | ACTGGGAAACACCGATGGAC |  |
| *G6Pase* | Forward | TCTGTCCCGGATCTACCTTG | 85 |
|  | Reverse | GAAAGTTTCAGCCACAGCAA |  |
| *PEPCK1* | Forward | ATCATCTTTGGTGGCCGTAG | 90 |
|  | Reverse | CATGGCTGCTCCTACAAACA |  |
| *GAPDH* | Forward | CATGGCCTTCCGTGTTCCTA | 104 |
|  | Reverse | CCTGCTTCACCACCTTCTTGAT |  |

**Text S1. HPLC Method Validation**

**(1) Specificity:** The blank solution was analyzed under the chromatographic conditions specified in the main text to evaluate baseline stability and detect potential interferences. The chromatogram demonstrated a stable baseline with no interfering peaks, thereby confirming the high specificity of the method (**Figure S1**).

**Figure S1** HPLC methodology specificity

**(2) Precision:** A 10 mg portion of the GSEA sample was accurately weighed and prepared into a solution, which was then injected six times consecutively. The relative peak areas of swertiamarin and gentiopicroside were recorded, and the corresponding RSD values were calculated to assess instrumental precision. The results indicated RSD values of 1.44% and 1.03% for swertiamarin and gentiopicroside, respectively, confirming the excellent precision of the instrument.

(3) **Repeatability**: Six individual portions of the GSEA sample, each weighing 10 mg, were independently prepared into solutions and analyzed. The relative peak areas of swertiamarin and gentiopicroside were recorded, and the corresponding RSD values were calculated. The results yielded RSD values of 1.49% and 1.64% for swertiamarin and gentiopicroside, respectively, indicating the good repeatability of the analytical method.

**(4) Stability**: A 10 mg portion of the GSEA sample was accurately weighed and prepared into a solution, which was then analyzed at 0, 2, 4, 8, 12, and 24 hours. The relative peak areas of swertiamarin and gentiopicroside were monitored over this period, and the RSD values were calculated. The results showed RSD values of 1.93% and 1.60%, respectively, indicating that the solution remained stable throughout the 24-hour testing period.

**(5) Linearity**: Equal volumes of swertiamarin and gentiopicroside standard solutions were mixed to prepare a combined solution with a concentration of 0.5 mg·mL^⁻¹^. This solution was then serially diluted to produce a range of concentrations, filtered through a 0.22 μm membrane, and analyzed according to the chromatographic conditions. Standard calibration curves were constructed by plotting the concentration (X-axis) against the relative peak area (Y-axis), and linear regression equations were derived. The results were as follows (**Figure S2**): swertiamarin, Y = 8715.9X + 15.19, R^2^ = 0.9999, linear range 7.81–500.00 μg·mL⁻¹; gentiopicroside, Y = 8207.6X + 2384.6, R^2^ = 0.9998, linear range 7.81–500.00 μg·mL^⁻¹^.

**Figure S2** Standard curves (A) swertiamarin; (B) gentiopicroside

**(6) Recovery**: Six portions of GSEA sample were spiked with two standard compounds at concentrations similar to those in the sample. The spiked solutions were prepared as outlined in the text and analyzed to measure the peak areas of swertiamarin and gentiopicroside. The concentrations were calculated using the standard calibration curves, and recovery rates were determined. The average recoveries were 101.74% for swertiamarin and 101.49% for gentiopicroside, with RSD values of 1.94% and 1.47%, respectively, confirming the reliable accuracy of the method for quantitative analysis.
